# The Barrier’s Heights and Its Inhomogeneities on Diamond Silicon Interfaces

**DOI:** 10.3390/ma15175895

**Published:** 2022-08-26

**Authors:** Szymon Łoś, Kazimierz Fabisiak, Kazimierz Paprocki, Wojciech Kozera, Tomasz Knapowski, Mirosław Szybowicz, Anna Dychalska

**Affiliations:** 1 Institute of Mathematics and Physics, Bydgoszcz University of Science and Technology, Profesora Sylwestra Kaliskiego 7, 85796 Bydgoszcz, Poland; 2 Department of Production Engineering Management, University of Bydgoszcz, Unii Lubelskiej 4c, 85059 Bydgoszcz, Poland; 3 Institute of Physics, Kazimierz Wielki University, Jana Karola Chodkiewicza 3, 85064 Bydgoszcz, Poland; 4 Department of Agricultural Chemistry, Faculty of Agriculture and Biotechnology, Bydgoszcz University of Science and Technology, Seminaryjna 5, 85326 Bydgoszcz, Poland; 5 Faculty of Material Engineering and Technical Physics, Poznan University of Technology, ul. Piotrowo 3, 60965 Poznań, Poland

**Keywords:** Raman spectroscopy, XRD, polycrystalline diamond film, p-PCD/n-Si heterojunction, I-V characteristics, energy barrier height, ideality factor

## Abstract

In this work, the electrical parameters of the polycrystalline diamonds’ p-PCD/n-Si heterojunction were investigated using temperature-dependent current–voltage (I-V) characteristics. In the temperature range of 80–280 K, the ideality factor (n) and energy barrier height (*φ_b_*) were found to be strongly temperature dependent. The *φ_b_* increases with temperature rise, while the n value decreases. The observed dependencies are due to imperfections at the interface region of a heterojunction and the non-homogeneous distribution of the potential barrier heights. Values of the *φ_b_* were calculated from I-V characteristics using the thermionic emission theory (TE). The plot of *φ_b_* versus 1/2 kT revealed two distinct linear regions with different slopes in temperature regions of 80–170 K and 170–280 K. This indicates the existence of a double Gaussian distribution (DGD) in heterojunctions. Parameters such as mean barrier heights ${\bar{\varphi }}_{b}$ and standard deviations σ were obtained from the plots linearization and read out from intercepts and slopes. They take values ${\bar{\varphi }}_{b}$ = 1.06 eV, σ = 0.43 eV, respectively. The modified Richardson plot is drawn to show the linear behavior in these two temperature ranges, disclosing different values of the effective Richardson constants (A*).

## 1. Introduction

The polycrystalline diamond layers obtained by the chemical vapor deposition methods (CVD) are attractive materials for the development of high-temperature, high-frequency, and high-power electronic devices, due to their significant properties, such as wide bandgap (5.45 eV at room temperature), high breakdown field (5–10 × 10^6^ V/cm), and high electron saturation velocity (v_s_ = 1.5–2.7 × 10^7^ cm·s^−1^) [1].

These bulk properties of diamond make it interesting for high-power and high-frequency electronic applications. Unipolar electronic devices, such as diodes or transistors, are of great interest, but their electronic performance is sensitive, among others, to surface terminations. Up to now, H- and O-terminated surfaces are the most frequently used for diamond power devices [2,3,4,5].

Although diamond exhibits several intriguing physical properties, due to a polycrystalline structure, the performance of CVD diamond-based devices may be hampered by a high concentration of structural defects and admixture of the non-diamond phase. We believe that the electrical conductivity of diamond layers will depend on a graphite-like admixture located mainly on the surface of microcrystallites and therefore on the size of the microcrystallites themselves. Additionally, the diamond layers obtained by CVD methods are generally highly hydrogenated, which also has a large impact on their electrical conductivity as well [6,7,8]. It is now well established that a hydrogen-terminated diamond surface exhibits p-type conduction in a subsurface layer without doping [8], with carrier density around 10^10^–10^13^ cm^–2^ [9]. H-terminated diamond has been extensively used, among others, for Schottky diodes construction. For the latter, H-diamond-based diodes have been demonstrated to have lower Schottky barrier height values in comparison to O-diamond-based Schottky diodes [10]. The electrical characteristics of the polycrystalline diamonds (PCD) p-PCD/n-Si heterojunction are determined by the energy barrier height at the interface and can depend on the surface preparation before the diamond synthesis process. An imperfect surface interface could cause heterogeneity, leading to non-ideal diode behavior [11,12]. This non-ideality includes the measurement of diode ideality factors (n) and barrier heights (BHs) at different temperatures.

The analysis of I-V-T characteristics of heterojunction shows an increase in barrier height and a decrease in the ideality factor, with a temperature increase [13]. The nature of such behavior has been successfully explained based on thermionic emission, (TE) theory with the assumption of a Gaussian distribution (GD) of barrier heights [14,15]. Such a distribution can be caused by inhomogeneities of the BHs at the p-PCD diamond/n-Si interface and can be explained by structural imperfection and by surface states. At lower temperatures, the current predominantly flows through regions with the lower BH, increasing the ideality factor, and at higher temperatures, region carriers can overpass a higher barrier, and the n tends to lower values.

Due to the modesty of scientific reports on the properties of n-Si/diamond heterojunctions based on undoped polycrystalline, we decided to undertake this type of research. In the present study, the forward bias I-V characteristics of p-PCD/n-Si heterojunction were measured in the temperature range of 80–280 K. The temperature-dependent barrier height and modified Richardson plot offer good straight lines in two temperature ranges, i.e., 80–170 K and 170–280 K. The resultant temperature dependences have been explained based on the existence of Gaussian distributions of the barrier heights around mean values due to the p-PCD/n-Si interface. The parameters of Gaussian distribution functions, i.e., mean value of barrier heights ${\bar{\varphi }}_{b}$ and σ were calculated based on I-V-T characteristics. The novelty of the present research is that it concerns the research on the properties of diamond/silicon heterojunctions based on undoped polycrystalline diamond layers, which act as a p-type semiconductor. The p-type electrical properties of this material result from the termination of diamond microcrystallites with hydrogen.

## 2. Materials and Methods

The undoped polycrystalline diamonds have been synthesized by using the hot filament chemical vapor deposition (HF CVD) method. The mixture of CH_3_OH/H_2_ was applied as a working gas. The films were synthesized on (111) oriented n-type Si substrate with a resistivity of 3.5 Ω cm. The apparatus’s reaction chamber consisted of a stainless steel tube with an internal diameter of 50 cm and was cooled by water. A tungsten filament with 2 mm distance from the substrate, heated up to 2100 °C, was used for thermal activation of the working gas mixture of methanol and hydrogen (CH_3_OH/H_2_ = 1 vol.%). The parameters of the growth process were as follows: the total pressure in the reaction chamber of p = 80 mbar, the substrate temperature of 1000 K, and the working gas flow rate of 100 sccm. The 99.99 purity methane and hydrogen gases were supplied by The Linde Gaz Group company. Before starting the diamond microcrystal growth process, the Si substrate was washed in acetone and then ethanol in an ultrasonic bath. To grow the continuous diamond layer, the substrate was mechanically polished to create surface defects, working as diamond nucleation centers, allowing the growth of a continuous polycrystalline layer.

The morphology of obtained diamond layers was studied by scanning electron microscopy (SEM) (JEOL JSM-820), Akishima, Japan. In turn, the phase purity was characterized by Raman spectroscopy. The Raman spectra were recorded at room temperature in backscattering geometry using Renishaw inVia Raman spectrometer (Renishaw confocal imaging systems), Great Britany, UK. The 488 nm argon laser line was used for excitation. The Raman measurements were made with an accuracy of 1 cm^−1^. The J-V-T measurements were performed in a configuration of the p-diamond/n-Si heterojunction. The electrical contacts were formed by depositing gold dots of 5 mm in diameter by thermal evaporation on the diamond surface and back of the Si substrate. More details can be found in our earlier paper [6].

## 3. Results

### 3.1. Surface Morphology Analysis

**Figure 1:**
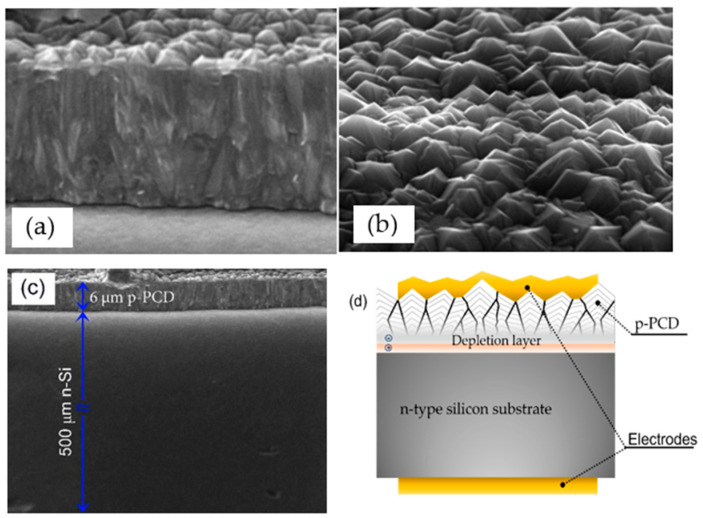
SEM images analysis of (**a**) cross-section of the diamond layer, (**b**) the surface morphology, (**c**) cross section of p-PCD/n-Si heterojunction, and (**d**) a two-dimensional schematic cross section of the heterojunction.

The SEM images of the studied diamond film are shown in Figure 1. At the bottom of the cross-section of diamond (Figure 1a), one can notice that there are distinct individual nanocrystallites that function as nucleation centers. After the nucleation stage, diamonds start to grow in the vertical direction along the fastest growing planes prevalent among the crystals. The resultant as-grown surface has remarkably high roughness due to the difference in heights of the growing columns, Figure 1b. The surface morphology shows an octahedral character. The cross section shown in Figure 1c discloses the PCD layer thickness and substrate as well. The scheme of the developed device is presented in Figure 1d.

### 3.2. Raman Spectroscopy and XRD

**Figure 2:**
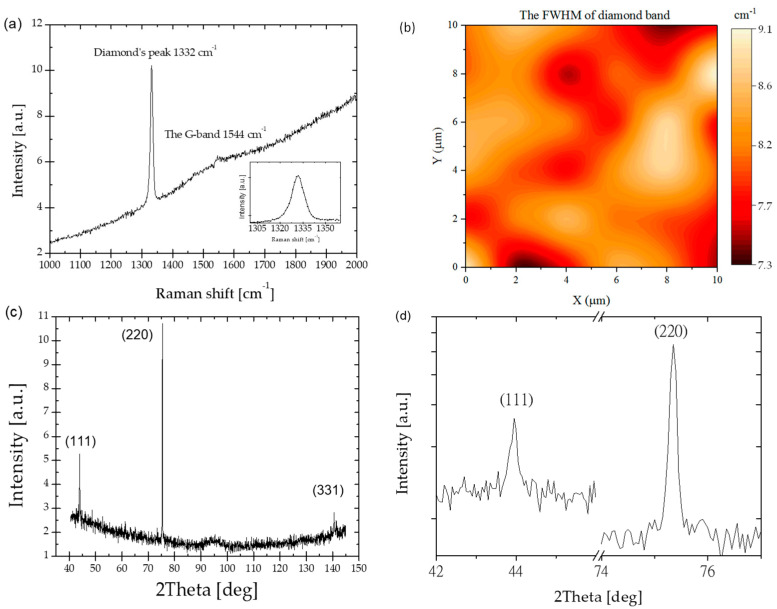
The structural analysis of diamond layers, (**a**) the Raman spectrum, in the insert, details of the diamond peak, (**b**) Raman’s mapping of the FWHM, (**c**) XRD diffractogram of the investigated diamond, and (**d**) details of the two most prominent diffraction peaks.

The Raman and XRD spectra of the diamond layer are shown in Figure 2. Figure 2a shows a strong diamond line peaked at 1332 cm^−1^, i.e., close to the theoretical value for the sp^3^-hybridized diamond carbon phase. The full width at half of the maximum (FWHM) for a well-crystallized diamond sample is 7.8 cm^−1^. This is an indication of the excellent quality of the diamond layer. Figure 2b shows the Raman’s map of the FWHM on an area of 100 µm^2^. There is a generally accepted convention that the value of the FWHM greater than 12 cm^−1^ means a poor quality diamond, and a value lower than 12 cm^−1^ of a diamond layer is considered good quality [16]. The Raman spectrum displays also a broad band peaked at 1544 cm^−1^ corresponding to the non-diamond carbon form with the predominant sp^2^-hybridization. As can be seen from Figure 2a, the Raman lines are superimposed on a broad luminescent background, indicating that the diamond layer is hydrogenated. The hydrogen in diamond layers is responsible for its p-type surface conductivity [17]. The purity of the diamond layers was estimated according to the procedure described in our earlier paper [18] and was equal to 98.8%. It means that diamond quality is superb because the sp^2^ admixture is below 1.2%.

The final morphology of the PCD layer depends on the preferential growth direction of microcrystals in the diamond layers. It is known that in different crystallographic orientations, the density of defects generated during the growth process depends on the growth direction [19]. The XRD diffraction pattern shows three main reflections from (111), (220), and (331) crystal planes. The diffraction patterns were compared with the JCPDS No. 6-0675 file. As can be seen in Figure 2d, the diffraction peaks are narrow. The FWHM is lower than 0.15 degrees. This confirms the good quality of the diamond layers. The texture analysis allows us to estimate the texture coefficients, which are as follows: 6%, 81.3%, and 12.7%, respectively, for Tc(111) Tc(220), and Tc(331). It means that our diamond layers have (220) preferential orientation.

### 3.3. Diodes Characteristics

**Figure 3:**
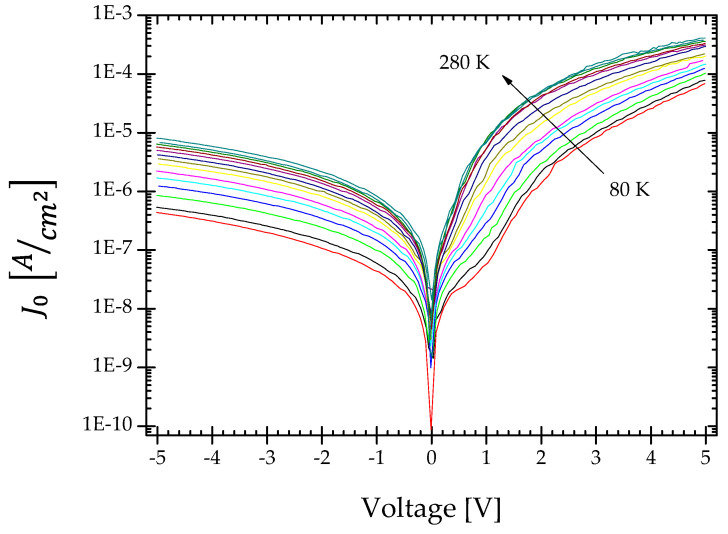
I-V-T characteristics of p-PCD/n-Si heterojunction.

The I-V characteristics of the p-PCD/n-Si heterojunction were measured in the temperature range from 80 to 280 K. They are shown in Figure 3. At 280 K, the heterojunctions showed a rectifying character. The rectification ratio is approximately about two orders of magnitude at bias voltages of ±3 V, and confirms that a p-n diode is formed at the interface of the p-PCD/n-Si. According to the TE theory, the forward current is a function of the voltage and temperature according to the equation

(1) $$J=A^{*}T^{2}\exp\left(-\frac{q\varphi_{b}}{kT}\right)\left[exp\left(\frac{qV}{nkT}\right)-1\right],$$

This equation can be simplified by removing the term (−1) which is justified when V≫3 kT/q, i.e., for *V* in the range of 0.1–0.4 V, which gives [20]

(2) $$J=J_{0}\left[exp\left(\frac{qV}{nkT}\right)\right]$$

where $A^{*}$ is the Richardson constant, T is the temperature, q is the electronic charge, $\varphi_{b}$ is the effective barrier height, k is the Boltzmann constant, V is the bias, and n is the ideality factor (for the ideal device n = 1), which is a dimensionless value. From the linear part of the plot of ln(*J*)–*V* usually in the range of 0–0.4 V, one can estimate the saturation current $J_{0}$ and the ideality factor n,

(3) $$n=\frac{q}{kT}\frac{dV}{d\left(lnJ\right)}$$

The value of the ideality coefficient is usually greater than one and may reach values even greater than 20 [21]. This is attributed to the presence of structural defects in the interfacial part of the heterojunction or series resistance, which influence the charges localization effect. Higher values reflect how much the energy of the biasing electric field has to be reduced in comparison to the thermal energy. However, a good and important explanation can be barrier inhomogeneities as well [22]. In particular, the large ideality factors already attracted attention very early [23,24] and are still being discussed. Previous explanations of high ideality factor values were based on trap-assisted tunneling or field-enhanced recombination via isolated point defect levels [25].

**Figure 4:**
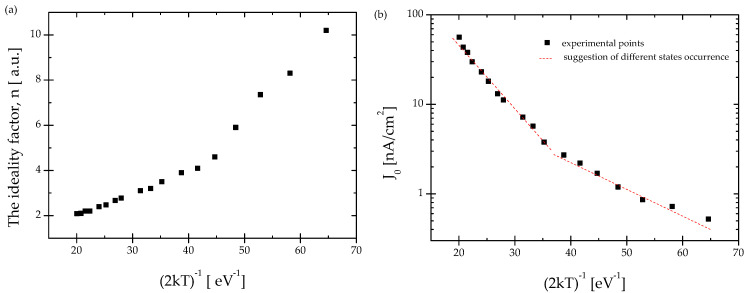
The temperature dependence of the experimental (**a**) ideality factor n, and (**b**) the reverse saturation current of the diode for Au/p-PCD/n-Si/Au structure.

The ideality factor and saturation current versus inverse temperature are shown in Figure 4a,b, respectively. As it is seen the ideality factor, n increases with decreasing temperature, while the reverse saturation current decreases. It is generally assumed that if the n is equal to one, the carriers freely cross the junction using the thermal diffusion process [26]. However, at a lower temperature, the process can be disturbed by the stronger localization effect on the defects, and this is reflected by the n rise. Figure 4b clearly shows the existence of two different values of the slopes of the graph $J_{0}$ vs. 1/2 kT in the range of lower (80–170 K) and higher (170–280 K) temperatures. This may indicate the occurrence of at least two types of interface states in the heterojunction [27].

The saturation current, *J*_0_(*T*) is given by the formula [26,28]:

(4) $$J_{0}=A^{*}T^{2}\exp\left(-\frac{\varphi_{b}}{kT}\right).$$

**Figure 5:**
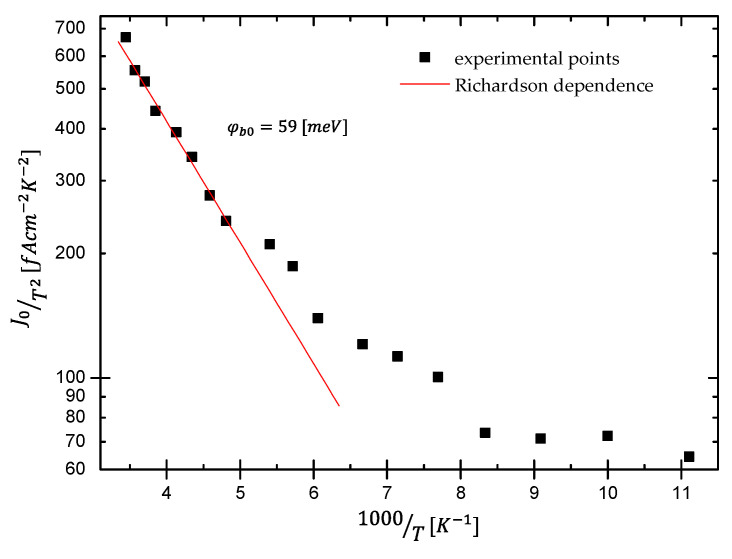
The conventional Richardson’s plot.

From the slope of Richardson’s plot, i.e., versus 1000/T presented in Figure 5, one can estimate the so-called zero-bias barrier height φ_b0_. As seen, the plot reveals behavior that the entire range of measured temperatures can be divided into two different regions: linear in higher temperature ranges and region where the dependence deviates from linearity assumed by the TE theory. Such discrepancy is associated with the potential barriers’ inhomogeneities. The interface is not atomically flat but rough, with the result of spatial fluctuations. Thus, it cannot be described by classical TE theory assuming the existence of a single potential barrier. However, it can be explained by the model proposed by Werner et al. [11]. The deviation of the Richardson plot from linearity indicates the temperature dependence of the BH and its inhomogeneity caused by potential fluctuations at the interface [29,30,31].

Solving Equation (4), the barrier heights can be derived from the relation [30]:

(5) $$\varphi_{b}=\frac{kT}{q}ln\left(\frac{A^{*}T^{2}}{J_{0}}\right).$$

**Figure 6:**
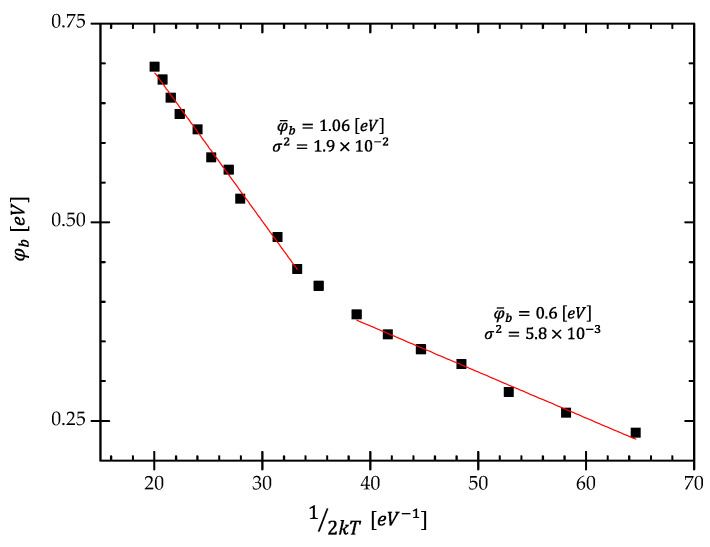
The variation of the barrier height $\varphi_{b}$ as a function of (1/2 kT) according to the Werner model.

The calculated values of *φ_b_* as a function of 1/2 kT are presented in Figure 6. The observed relation suggests the existence of two different barrier height distributions [32]. The increase in *φ_b_*, with the temperature rise, is related to the presence of trap states in the interfacial layer. Due to the negative value of electrons’ affinity for the diamond structure [31], thermally emitted charges from the n-Si surface create a depletion layer with higher potential. According to Sullivan et al. [33], the barriers consist of laterally inhomogeneous patches of different barrier heights. The patches with lower barrier height yield a larger ideality factor and vice versa. There is indicated the temperature dependence of a density of interface states [6]. The inhomogeneity due to potential fluctuations can be described using the Gaussian distribution function of the barrier height around an average value. Hence, the total saturation current $J_{0}$ can be expressed by the formula [13]

(6) $$J_{0}=A^{*}T^{2}\int_{0}^{\infty }A\left(\varphi_{b}\right)\exp\left(-\frac{q\varphi_{b}}{kT}\right)d\varphi_{b}$$

where $A(\varphi_{b})$ is a distribution function that describes the Schottky barrier inhomogeneities. Taking into account that the barrier variability can be approximated with two lines, in Figure 6, the distribution function can be assumed to be the sum of two Gaussians as is described in the following relation:

(7) $$A(\varphi_{b})=\frac{A_{1}}{\sigma_{1}\surd 2\pi }exp\left[-\frac{{\left(\varphi_{b}-{\bar{\varphi }}_{b1}\right)}^{2}}{2\sigma_{1}^{2}}\right]+\frac{A_{2}}{\sigma_{2}\surd 2\pi }exp\left[-\frac{{\left(\varphi_{b}-{\bar{\varphi }}_{b2}\right)}^{2}}{2\sigma_{2}^{2}}\right].$$

The parameters ${\bar{\varphi }}_{bi}$ and $\sigma_{i}^{2}$ (*i* = 1,2) of distribution functions can be obtained using the formula [22]

(8) $$\varphi_{b}={\bar{\varphi }}_{bi}-\frac{q\sigma_{i}^{2}}{2kT}.$$

The plot of $\varphi_{b}$ shown in Figure 6 allows estimating two parameters of the distribution function, average values ${\bar{\varphi }}_{bi}$ of the BH and the standard deviation *σ_i_*. They are as follows: BHs 0.6 eV and 1.06 eV and σ 0.0762 eV and 0.43 eV for lower 80 K–170 K and higher 170 K–280 K temperature ranges, respectively. The good fit of the experimental points by straight lines presented in Figure 6 confirms the hypothesis of the DGD of potential BHs.

Combining Equations (4) and (8), we obtain

(9) $$\ln\left(\frac{J_{0}}{T^{2}}\right)-\frac{q^{2}\sigma_{i}^{2}}{2k^{2}T^{2}}=ln\left(AA^{*}\right)-\frac{{\bar{\varphi }}_{bi}}{kT}.$$

**Figure 7:**
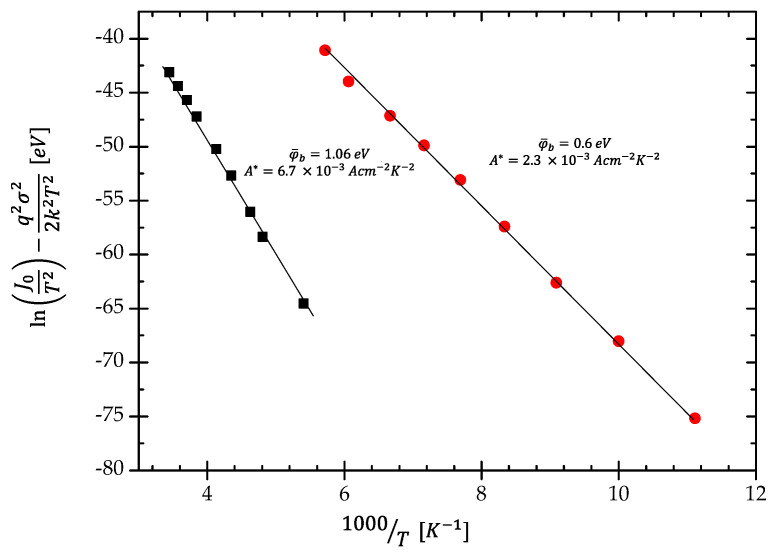
The modified Richardson’s plot.

This equation called the modified Richardson’s relation allows the estimation of the Richardson’s constant A* and amplitudes of the double Gaussian distribution function as well. Its plot versus inverse temperature produces again the straight line with the slope of the average barrier’s height. Yet, the intercept at the ordinate designates the A* value. Using Equation (9) to both temperature ranges gives the A* as 6.7 × 10^−3^ A cm^−2^ K^−2^ and 2.3 × 10^−3^ A cm^−2^ K^−2^, respectively, to higher and lower temperature ranges. Results are shown in Figure 7. Two different values of the Richardson constants result from the fact that, depending on the temperature range, we have two different Gaussian barrier potential distributions. The obtained values of Richardson’s constants are much smaller than the theoretical one of 90 A cm^−2^ K^−2^ [34]. The values of the Richardson’s constants reported in the literature [4,35,36,37] are in the wide range of 1.0995–1.9 × 10^−9^ A cm^−2^ K^−2^. To date, the problem of the measured Richardson’s constant values for several types of junctions has not been properly explained. However, to obtain a good fit, the Gaussian amplitudes need to be arbitrarily chosen. They undergo the normalization condition *A*_1_(*φ_b_*_1_) + *A*_2_(*φ_b_*_2_) = 1. Finally, estimated values are 0.64 and 0.36, respectively, for both Gaussian functions at higher and lower temperature ranges.

Based on the research carried out, it can be said that the ideality factor increases with the temperature decrease, while the height of the potential barrier decreases. At the higher temperature range, where the n is close to one, charges are subjected to a thermally diffusive process with a higher value of average potential and emission Richardson’s constant as well. A gradual temperature decrease results in parameter change, presumably due to the charges localization effect at interstitial defects. In the case of this study, it was found a 170 K temperature for the abrupt parameters change. Below and above this temperature, the averaging needs to be calculated around other values due to the impact of the stronger influence of the localization effect at lower temperatures. Localized charges have an influence on each aspect of current carriers’ transport through the heterojunction: the ideality factor, barrier’s height, and barrier homogeneity, as well as the series resistance R_s_ [6,7].

## 4. Conclusions

In the present work, the heterojunctions of p-type PCD/n-Si were developed. PCD film was grown by the HF CVD method, while the n-type Si substrate is a commercially available single crystal. The structural properties of the diamond layer were characterized by SEM, Raman spectroscopy, and XRD. The J-V-T characteristics were measured in the temperature range of 80–280 K. From the J-V-T curves, the junction parameters, i.e., the ideality factor n and the height of the potential barrier $\varphi_{b}$, were computed using the TE theory. Through the TE theory, the value for the n varies from 2.7 at 280 K to 8.7 at 80 K, while $\varphi_{b}$ increases with temperature. Due to the assumed inhomogeneity of the heights of the potential barriers, the range of the temperature changes was divided into two ranges. The obtained average values of ${\bar{\varphi }}_{b}$ in lower 80 K–170 K, and higher 170 K–280 K temperature ranges are 0.6 eV and 1.06 eV, respectively. The averaging was performed by the double Gaussian distribution function. Its parameters are as follows: amplitudes of 0.64 and 0.36 and standard deviations of 0.43 eV and 0.0762 eV, respectively, to higher and lower temperature ranges. This procedure allows the linearization of the modified Richardson’s relation and the estimation of Richardson’s constant A*. They take values of 6.7 × 10^−3^ A cm^−2^ K^−2^ and 2.3 × 10^−3^ A cm^−2^ K^−2^, respectively, to higher and lower temperature ranges. In our opinion, the division in two temperature ranges is caused by the charge localization influence on interfacial defects. This effect is stronger at lower temperatures.

## Data Availability

Data are not available.
